# Neonatal Sleeve Gastrectomy for Multiple Gastric Perforations: A Case Report

**DOI:** 10.21699/jns.v5i3.355

**Published:** 2017-01-01

**Authors:** Francisco Reyna-Sepulveda

**Affiliations:** General Surgery Department, University Hospital “Dr. José Eleuterio González”, Autonomous University of Nuevo León.

**Keywords:** Neonate, Gastric, Perforation, Gastric sleeve

## Abstract

Neonatal gastric perforation (NGP) may be spontaneous, secondary to necrotizing enterocolitis (NEC), or due to distal obstruction. A 27-week old premature male newborn presented with pneumoperitoneum. A single perforation of stomach was found at surgery. Primary repair and gastrostomy were performed. On fifth postoperative day, pneumoperitoneum was again detected. At reoperation, multiple gastric perforations of the greater curvature were found. Sleeve gastrectomy was performed. The patient responded well to the treatment.

## CASE REPORT

A premature 27-week old male, weighing 1110grams, born to a 28-year-old mother required orotracheal intubation for poor respiratory efforts owing to prematurity. In Neonatal ICU, the newborn had an episode of cardiac arrest which responded to cardiopulmonary resuscitation within a minute. Next day enteral feeding was started but he developed gastric distension, and irritability. An orogastric tube was placed which drained bile. An abdominal x-ray demonstrated pneumoperitoneum. At laparotomy, a single gastric perforation of the posterior wall of the body of stomach was found which was managed with primary repair and gastrostomy. On fifth postoperative day, abdominal distension was found and an abdominal x-ray again showed pneumoperitoneum. At reoperation, multiple gastric perforations of the greater curvature of stomach were found (Fig. 1). A sleeve gastrectomy was performed by initially identifying the pylorus of the stomach and the greater curve. The greater omentum is entered and the greater curvature of the stomach is then dissected free from the omentum and the short gastric blood vessels. Sleeve gastrectomy was then done using a two-layer continuous repair starting 1cm from the pylorus and proceeding to the Angle of His. An upper gastrointestinal contrast study was performed on the 7th postoperative day demonstrated no leak (Fig. 2). The patient remained stable and tolerated nasogastric feeding until follow-up on the 15th postoperative day. Histopathology from the gastric resection reported hemorrhagic necrosis and a nonspecific acute inflammatory process without anatomic alterations.

**Figure:**
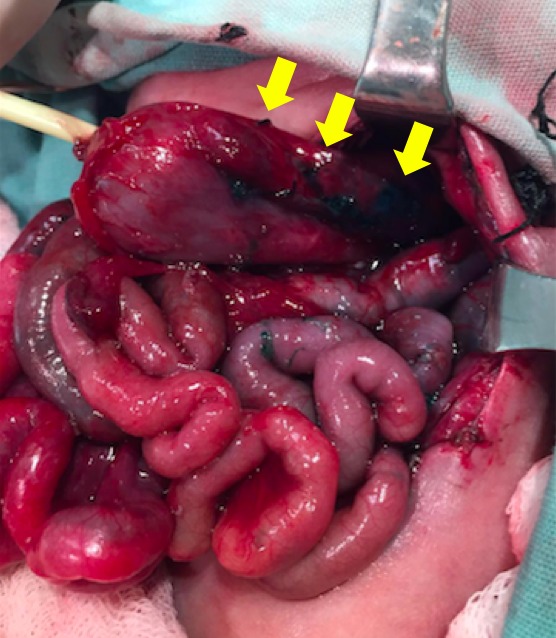
Figure 1: Multiple gastric perforations shown with arrows.

**Figure:**
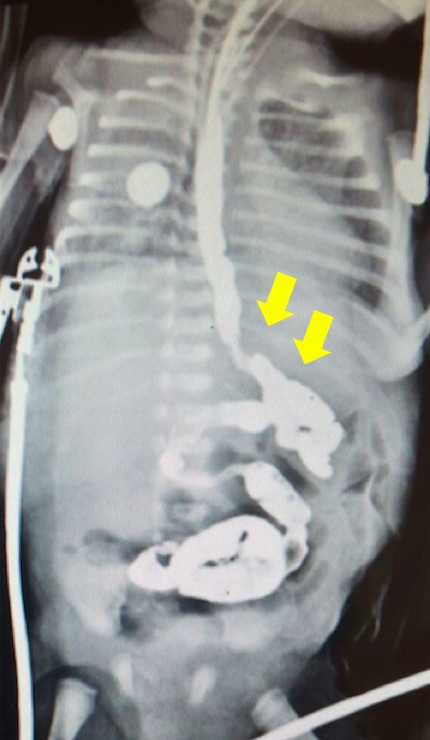
Figure 2: No leakage found.

## DISCUSSION

Gastric perforation can be caused by increased gastric pressure by vigorous resuscitation and secondary to a big distal fistula in case of esophageal atresia with tracheoesophageal fistula, vigorous nasogastric tube insertion, distal obstruction, NEC, or spontaneous [1-4]. Management of gastric perforation includes primary closure with or without gastrostomy.[5] In our case, due to the multiple lesions located on the greater curvature a sleeve gastrectomy was performed. We believe that a gastric sleeve can be selected as a therapeutic option when there is adequate vasculature in the lesser curvature as occurred in our patient. Sleeve gastrectomy preserves the gastric chamber and maintains esophageal, gastric and duodenal continuity without total gastrectomy which has high morbidity and mortality. To the best of our knowledge, this is first report of sleeve gastrectomy for multiple gastric perforations in neonates.

## Footnotes

**Source of Support:** Nil

**Conflict of Interest:** Nil
